# Real-World Validation of the AI-Enabled Vectorcardiographic CSG-Index for Obstructive Coronary Artery Disease: The VECTOR-CAD Study

**DOI:** 10.3390/diagnostics16203266

**Published:** 2026-10-09

**Authors:** Muhammed Esad Çekin, Halil Gökkuş

**Affiliations:** 1 Department of Cardiology, Faculty of Medicine, Bolu Abant İzzet Baysal University, Bolu 14030, Türkiye; 2 Department of Radiology, Bolu İzzet Baysal State Hospital, Bolu 14300, Türkiye; halilefegokkus@gmail.com

**Keywords:** artificial intelligence, vectorcardiography, Cardisiography, coronary artery disease, diagnostic accuracy, exercise electrocardiography

## Abstract

**Background/Objectives:** Cardisiography is an artificial intelligence-enabled resting vectorcardiography (AI-VCG) system, but evidence for its accuracy in coronary artery disease (CAD) remains limited. We evaluated the AI-VCG-derived CSG-index for obstructive CAD detection, association with stenosis severity, incremental discrimination beyond clinical variables, and concordance with exercise electrocardiography (ECG). **Methods:** In this two-centre retrospective diagnostic-accuracy study, 721 Cardisiography records from January 2023–December 2025 were screened and 224 patients were included. Invasive coronary angiography (ICA) was the primary reference (*n* = 172); coronary computed tomography angiography (CCTA) contributed only reference-negative cases (*n* = 52) to the hierarchical cohort. Obstructive CAD was defined as ≥50% stenosis and CSG-index ≥ −0.27 as positive. Primary analysis assessed obstructive CAD discrimination in the ICA cohort; secondary analyses assessed severe CAD (≥70%), hierarchical-cohort performance, stenosis severity, incremental value beyond available clinical variables and exercise ECG concordance. **Results:** Obstructive CAD was present in 76/172 patients. In the ICA cohort, CSG-index area under the curve (AUC) was 0.820, with 80.3% sensitivity and 78.1% specificity; AUCs were 0.843 in the hierarchical cohort and 0.785 for severe CAD. CSG-index increased across stenosis classes. After adjustment for available clinical variables, it remained independently associated with obstructive CAD and increased model AUC from 0.806 to 0.886. In the exploratory exercise ECG subgroup (*n* = 83), agreement was 66.3%, with fewer false-positive CSG-index classifications. **Conclusions:** The locked CSG-index showed balanced performance for obstructive CAD, provided graded information across stenosis classes, and added discrimination beyond available clinical variables. These findings support prospective multicentre evaluation as a complementary non-invasive resting test.

## 1. Introduction

Coronary artery disease (CAD) remains a leading cause of morbidity and mortality worldwide and imposes substantial healthcare costs [1,2,3]. Chronic coronary syndromes contribute considerably to this burden and to outpatient cardiology activity, while the identification of obstructive CAD informs the intensity of preventive treatment, the need for further anatomical or functional assessment, and consideration of revascularisation [4]. Contemporary diagnostic pathways integrate symptoms and cardiovascular risk factors with exercise electrocardiography, coronary computed tomography angiography (CCTA), functional imaging, and invasive coronary angiography (ICA); test selection depends on clinical likelihood, patient characteristics, local availability, and clinical judgement [4].

In routine clinical practice, however, diagnostic pathways are heterogeneous. Patients may undergo exercise ECG, myocardial perfusion scintigraphy (MPS), or CCTA, or proceed directly to ICA, sometimes after one or more of these tests. Although these modalities are well established, they may require radiation exposure, iodinated contrast media, exercise or pharmacological stress, invasive catheterisation, or specialised infrastructure; each also has specific limitations. This heterogeneity and these constraints sustain interest in brief, inexpensive, radiation-free, and minimally operator-dependent resting tools that may provide complementary information before more resource-intensive investigations.

Although widely available, conventional resting 12-lead ECG may be normal or show only non-specific abnormalities in chronic CAD; routine interpretation may therefore miss subtle multidimensional signal patterns. Vectorcardiography partly addresses this limitation by representing cardiac electrical activity as three-dimensional vector loops rather than scalar leads, thereby preserving spatial information on depolarisation and repolarisation trajectories [5,6]. Machine-learning methods can extract high-dimensional features from these recordings that are not accessible through visual interpretation. Artificial intelligence (AI) applied to resting ECG has been reported to detect angiography- or CCTA-defined coronary disease and provide discrimination beyond clinical variables [7,8,9,10].

Cardisiography is an AI-enabled vectorcardiographic (AI-VCG) system that generates a continuous CSG-index from a resting recording. Earlier studies of cardiogoniometry, a precursor vectorcardiographic method, supported the feasibility of detecting CAD at rest but were heterogeneous in their populations, algorithms, stenosis thresholds, and designs [5,11]. Subsequent AI-VCG studies reported promising findings, but the available evidence came from an algorithm-development cohort [12], MPS-based functional validation [13], or population-based cardiovascular risk assessment without an anatomical endpoint [14]. Accordingly, real-world evidence for the locked CSG-index at its prespecified threshold against anatomically defined obstructive CAD remains limited.

In clinical practice, anatomical testing is applied selectively: some patients undergo ICA, whereas others undergo CCTA, and patients with reassuring negative CCTA findings generally do not proceed to invasive catheterisation. Consequently, validation cohorts restricted to patients referred for ICA may be susceptible to referral and disease-spectrum effects and may not adequately represent routine outpatient practice. Although the use of different reference-standard tests may influence estimates of diagnostic accuracy [15,16], evidence from such mixed diagnostic pathways remains limited. To address this gap, we retained the ICA cohort for the primary analysis and constructed a hierarchical anatomical cohort in which patients without ICA were included as reference-negative cases only when CCTA demonstrated either no CAD or non-obstructive disease. This cohort was designed as a pragmatic robustness analysis rather than an alternative primary reference standard.

The Vectorcardiography for Coronary Artery Disease (VECTOR-CAD) study was a two-centre, retrospective, real-world diagnostic-accuracy study of the AI-VCG-derived CSG-index in the routine assessment of suspected CAD. The CSG-index was locked, with no study-specific retraining or recalibration; the manufacturer-defined cut-off of −0.27 was prespecified and not optimised using the study data. The primary objective was to assess the discrimination of the continuous CSG-index and its diagnostic accuracy at this threshold for ICA-defined obstructive CAD (≥50% stenosis). Secondary objectives were to assess discrimination for severe CAD (≥70% stenosis) and in the hierarchical cohort; evaluate its ordered association with stenosis class; determine its incremental discrimination beyond available demographic and clinical risk variables and exploratory consistency by sex; and evaluate its performance in the exercise ECG and MPS subgroups, including concordance with exercise ECG. We hypothesised that the CSG-index would be associated with obstructive CAD, increase across stenosis classes, and provide information beyond clinical characteristics.

## 2. Materials and Methods

### 2.1. Study Design, Setting, and Participants

The VECTOR-CAD study was a two-centre, retrospective, observational diagnostic-accuracy study using routinely collected clinical records from adults who underwent resting Cardisiography between 1 January 2023 and 31 December 2025 in the cardiology departments of Bolu Abant İzzet Baysal University Training and Research Hospital and Bolu İzzet Baysal State Hospital, Bolu, Türkiye. In all included patients, Cardisiography was performed during the initial outpatient evaluation and before the anatomical reference examination. The results were available to the treating cardiologists; downstream anatomical testing was selected during routine care according to clinical assessment, and no study protocol linked referral decisions to the CSG-index.

**Figure 1:**
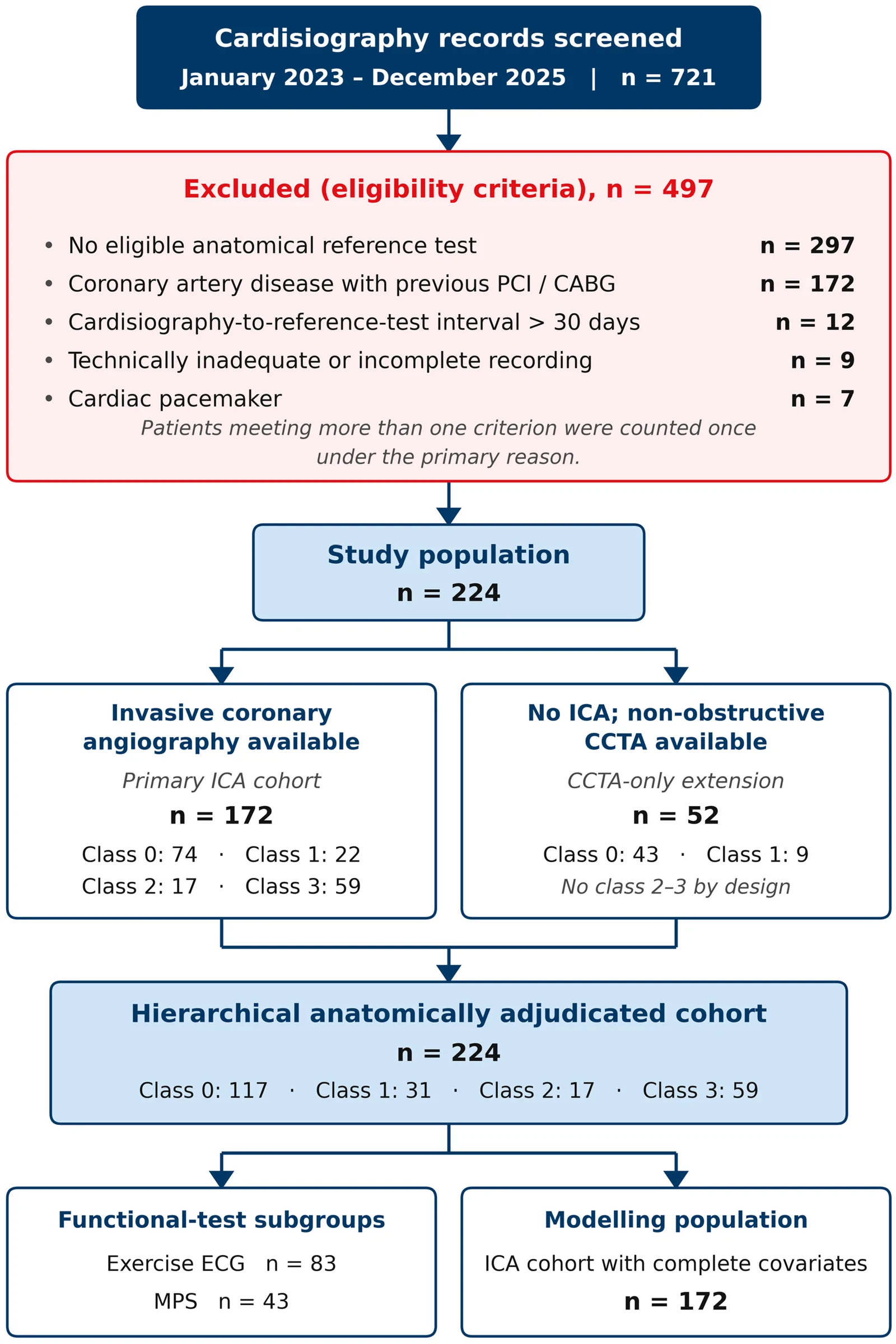
Participant flow diagram. The diagram shows the Cardisiography records screened between January 2023 and December 2025 (*n* = 721), the excluded cases and reasons for exclusion (*n* = 497), and the resulting study population (*n* = 224). Patients meeting more than one exclusion criterion were counted only once according to the primary reason for exclusion. The primary ICA cohort included all eligible patients with invasive anatomical adjudication. Patients without ICA contributed to the hierarchical anatomically adjudicated cohort only as reference-negative cases when CCTA demonstrated class 0 or class 1 disease. All patients with class 2–3 disease on CCTA (*n* = 21) subsequently underwent ICA; no positive or indeterminate CCTA examination remained without invasive verification. Functional-comparator and multivariable-modelling analyses were restricted to the primary ICA cohort. Stenosis classes were defined as follows: class 0, no stenosis; class 1, 1–49%; class 2, 50–69%; and class 3, ≥70%. Abbreviations: CABG, coronary artery bypass grafting; CCTA, coronary computed tomography angiography; ECG, electrocardiography; ICA, invasive coronary angiography; MPS, myocardial perfusion scintigraphy; PCI, percutaneous coronary intervention.

Adults aged ≥ 18 years were eligible if they underwent resting Cardisiography during routine outpatient evaluation and had an adjudicable prespecified anatomical outcome. Exclusion criteria were prior percutaneous coronary intervention (PCI) or coronary artery bypass grafting (CABG); a cardiac pacemaker, which precluded Cardisiography analysis; technically inadequate or incomplete Cardisiography recordings; absence of ICA or an eligible negative CCTA examination; and a Cardisiography-to-reference-test interval > 30 days. No prospective sample-size calculation was performed because the study used the available retrospective cohort; all records meeting the prespecified analytical eligibility criteria were included. The study is reported in accordance with the STARD 2015 guidelines; the completed checklist is provided in the Supplementary Materials. Participant flow and exclusion reasons are shown in Figure 1.

### 2.2. Index Test: Cardisiography/AI-Enabled Vectorcardiography

Cardisiography (Cardisio GmbH, Frankfurt am Main, Germany) is an AI-enabled direct vectorcardiographic system. Resting recordings lasting approximately four minutes were acquired using five electrodes in a predefined thoracic configuration, without stress induction, contrast media, ionising radiation, or radioactive agents. Three signal electrodes and one ground electrode were positioned on the anterior chest wall, while a fourth signal electrode was placed on the lower left side of the back to capture the third spatial dimension. Bipolar leads derived from these electrodes were orthogonally projected into a Cartesian coordinate system, providing a direct three-dimensional representation of cardiac depolarisation and repolarisation rather than a post hoc reconstruction from a conventional 12-lead ECG, as previously described [5,12]. Recordings were obtained by trained clinical personnel as part of routine care. The device software automatically performed signal acquisition and quality control; recordings that did not meet the manufacturer’s signal-quality requirements were excluded as technically inadequate.

Signals underwent automated cloud-based analysis using a supervised machine-learning model, without human interpretation or adjudication. The model architecture was described in a previously published implementation. In that implementation, the bipolar thoracic leads were transformed into an orthogonal three-dimensional (x, y, z) derivation, and 290 parameter series describing QRS- and T-wave timing, vector-loop morphology, electrical energy density, and vector magnitudes and directions were derived from each recording. Eight summary statistics for each parameter series generated 2320 candidate features, from which 27 inputs were selected based on discriminative performance and inter-feature correlation. These inputs were fed into an ensemble of five feed-forward multilayer perceptrons, each comprising 27 input neurons, two hidden layers with 20 and 22 neurons, and one output neuron [12].

The routine clinical report displays the CSG-index together with the QRS angle, T angle, spatial QRS–T angle, QRS–T superposition, T magnitude, Scatter QRS, and Scatter T; however, these reported parameters should not be interpreted as the complete set of model inputs or as measures of feature importance. The preprocessing, signal-processing, and feature-extraction procedures of the current system, the identities of the selected inputs, model weights, and source code are proprietary and were unavailable to the investigators. Therefore, the description above refers to the published implementation, and its detailed component-level correspondence with the current implementation could not be independently verified.

The original algorithm-development cohort comprised patients from three German centres, and neither of the two participating centres was part of that cohort [12]. The Cardisiography system was used in its commercially available configuration for routine clinical practice. The system software underwent updates during the study period; however, according to clarification provided by the manufacturer, these changes did not affect the mathematical calculation or interpretation of the CSG-index. Accordingly, in this study, the term “locked” refers to the CSG-index: its calculation remained unchanged throughout the study period, and no study-specific retraining, recalibration, tuning, feature modification, centre-specific adaptation, or threshold optimisation was performed by the investigators. Because the commercial reports did not display a version identifier and the software version used for each recording was not documented, version numbers could not be reported.

The prespecified index-test output was the CSG-index, a continuous algorithmic score ranging from −1 to +1, with higher values denoting more abnormal patterns. The manufacturer-defined cut-off of −0.27, documented in the product guidance and routine clinical reports, was selected by the manufacturer on the basis of the balance between sensitivity and specificity observed during algorithm development. The cut-off was neither derived nor re-estimated from the present data; it was prespecified in the study protocol submitted for ethics approval before data analysis and remained unchanged throughout the study. Values ≥ −0.27 were classified as index-test positive. Although the system report also included conventional electrocardiographic and vectorcardiographic parameters, these were not analysed; the study was restricted to validating this single clinically interpretable output.

### 2.3. Anatomical Reference Standard and Analysis Cohorts

Invasive coronary angiography (ICA) was interpreted by an interventional cardiologist with 13 years of experience in coronary angiography. The greatest luminal diameter reduction in any major epicardial coronary artery, estimated visually without quantitative coronary angiography, defined patient-level stenosis severity as class 0, no stenosis; class 1, 1–49% stenosis; class 2, 50–69% stenosis; or class 3, ≥70% stenosis. The primary diagnostic target, obstructive CAD, comprised classes 2–3 (≥50% stenosis). Severe CAD was the secondary target and comprised class 3 (≥70% stenosis), with classes 0–2 forming the reference-negative group.

CCTA examinations were performed using a Revolution EVO CT system (GE HealthCare, Chicago, IL, USA) and interpreted by a radiologist with 11 years of experience. In the prespecified hierarchical anatomical cohort, ICA findings took precedence; patients without ICA were included only when CCTA showed class 0 or 1 disease and were classified as reference-negative. Accordingly, all reference-positive cases were confirmed by ICA, whereas negative status could be established by ICA or CCTA. No patient with obstructive or indeterminate CCTA findings remained without invasive verification: all 21 patients with class 2–3 disease on CCTA subsequently underwent ICA and were classified according to the ICA findings, and no CCTA examination was indeterminate.

Cardisiography reports, including the numerical CSG-index and its positive or negative classification, were accessible in the clinical records when the anatomical examinations were interpreted. Whether each reader reviewed the Cardisiography report in every case could not be verified retrospectively; the readers were therefore considered not blinded to the index-test results or to other available clinical information. No central core-laboratory assessment or independent duplicate adjudication was performed. The primary ICA cohort comprised all eligible patients who underwent ICA and was used for the primary and severe-CAD endpoints, baseline comparisons, stenosis-severity analyses, functional-comparator analyses, and multivariable modelling.

### 2.4. Clinical Variables and Functional Comparator Tests

Age, sex, height, weight, current smoking, diabetes mellitus, hypertension, and hyperlipidaemia were extracted from the clinical records. Body mass index (BMI) was calculated as weight in kilograms divided by height in metres squared. Current smoking and comorbidities were coded as binary variables according to their documented clinical status. These variables were prespecified for the clinical multivariable model; no automated variable-selection procedure was used. Symptom characteristics, resting ECG findings, and pre-test likelihood estimates were not systematically recorded and were therefore not included.

Exercise ECG and MPS were performed for routine clinical indications rather than under a study-specific protocol. Their final clinical interpretations were recorded as binary positive or negative results, and analyses were restricted to patients with available results in the ICA cohort. Because each comparator yielded only a single binary operating point, an area under the receiver operating characteristic curve (AUC) was not estimated for either test; AUCs were calculated for the continuous CSG-index within each subgroup. Agreement between the dichotomised CSG-index and exercise ECG was summarised using four concordance categories. Functional-comparator analyses were exploratory and were not designed as prospective head-to-head comparisons or assessments of clinical utility.

### 2.5. Statistical Analysis

Continuous variables were summarised as median (interquartile range) and compared using the Mann–Whitney U test; categorical variables were summarised as count (percentage) and compared using Fisher’s exact test. Sensitivity, specificity, positive and negative predictive values, and accuracy at the prespecified CSG-index threshold were reported with Wilson 95% confidence intervals (CIs). Discrimination of the continuous CSG-index was quantified by the area under the curve (AUC) with DeLong 95% CIs. In a post hoc exploratory analysis of potential referral-related differences in the reference-negative spectrum, ICA-verified and CCTA-only reference-negative patients were compared using Fisher’s exact test for CSG-index positivity and the Mann–Whitney U test for continuous CSG-index values.

Two prespecified multivariable logistic models were fitted in the primary ICA cohort. The Clinical model included age, sex, body mass index, current smoking, diabetes mellitus, hypertension, and hyperlipidaemia; the Clinical + CSG-index model additionally included the CSG-index, standardised to the ICA-cohort mean and standard deviation. Maximum-likelihood estimates were reported as odds ratios with 95% Wald CIs, with the CSG-index expressed per 1-standard-deviation increase. Incremental discrimination was evaluated using the likelihood-ratio test and a paired DeLong comparison of AUCs; optimism-corrected AUCs were estimated using 2000 bootstrap resamples. These secondary models were not intended as deployable clinical risk-prediction tools.

Associations between the CSG-index and ordered ICA classes were assessed using Spearman’s rank correlation and a one-sided Jonckheere–Terpstra permutation test with 49,999 permutations. A proportional-odds ordinal logistic model estimated the common odds ratio per one-unit increase in the CSG-index; the proportional-odds assumption was evaluated using the Brant test. Sex-stratified AUCs and testing of the CSG-index × sex interaction in the fully adjusted model were exploratory. As a post hoc sensitivity analysis, 95% confidence intervals for the sex-specific AUCs were additionally estimated using 2000 nonparametric bootstrap resamples stratified by reference status within each sex; because the locked index was not fitted to the study data, no optimism correction was applied to these estimates.

Within functional-test subgroups, metrics for the binary comparators and the dichotomised CSG-index were reported with Wilson 95% CIs, and AUCs for the continuous CSG-index were reported with DeLong 95% CIs. Paired comparisons of overall classification, sensitivity, and specificity between the dichotomised CSG-index and exercise ECG were performed using exact McNemar tests. To evaluate selection into the exercise ECG subgroup, propensity scores for exercise ECG availability were estimated post hoc in the primary ICA cohort using logistic regression that included age, sex, body mass index, current smoking, diabetes mellitus, hypertension, and hyperlipidaemia. Stabilised inverse-probability-of-testing weights were applied, and covariate balance was assessed using absolute standardised mean differences (SMDs) before and after weighting. Propensity-score overlap, weight distribution, and effective sample size were examined, and weighted prevalences of obstructive and severe CAD were compared, with 95% CIs derived from 2000 bootstrap resamples in which the propensity model was re-estimated.

The primary ICA cohort had no missing index-test, reference-standard, or modelling-variable data and no indeterminate CSG-index results; therefore, no imputation was performed. Exercise ECG and MPS analyses used available cases, with denominators reported explicitly. Except for the one-sided ordered trend test, all tests were two-sided, with *p* < 0.05 considered significant. Multiplicity adjustment was not applied. Secondary analyses were considered supportive, whereas subgroup, functional-comparator, and post hoc analyses were considered exploratory. Analyses used Python 3.12.13 (Python Software Foundation, Wilmington, DE, USA), pandas 2.2.3, NumPy 2.3.5, SciPy 1.17.0, statsmodels 0.14.6, scikit-learn 1.8.0, and Matplotlib 3.10.8.

### 2.6. Ethics and Data Governance

The study was approved by the Bolu Abant İzzet Baysal University Non-Interventional Clinical Research Ethics Committee (Decision No. 2026/153; approval date: 7 April 2026) and was conducted in accordance with the Declaration of Helsinki. Because this was a retrospective archive analysis of routine outpatient examinations using de-identified clinical data, written informed consent for participation was not required in accordance with national legislation and institutional requirements. Direct identifiers were removed before analysis, and only de-identified data were used for statistical evaluation.

## 3. Results

### 3.1. Cohort Composition and Baseline Characteristics

Of 721 Cardisiography records screened, 497 were excluded, leaving 224 patients: 172 in the primary ICA cohort and 52 in the CCTA-only extension; the latter contributed exclusively to the hierarchical analyses (Figure 1). ICA classes 0–3 comprised 74, 22, 17, and 59 patients, respectively; 76/172 (44.2%) had obstructive CAD (≥50% stenosis), and 59/172 (34.3%) had severe CAD (≥70% stenosis). In the hierarchical cohort, 76/224 (33.9%) patients had obstructive CAD, whereas 148/224 (66.1%) had non-obstructive CAD (Supplementary Table S1). In the ICA cohort, the median age was 60.0 years (interquartile range [IQR], 53.0–68.0), and 75/172 (43.6%) patients were women. Compared with patients without obstructive CAD, those with obstructive CAD were older (63.0 [58.0–70.3] vs. 57.0 [51.0–65.0] years; *p* < 0.001), less often women (34.2% vs. 51.0%; *p* = 0.031), and more frequently had diabetes mellitus (43.4% vs. 26.0%; *p* = 0.023) and hypertension (82.9% vs. 43.8%; *p* < 0.001). Body mass index, current smoking, and hyperlipidaemia did not differ significantly between groups (Table 1).

### 3.2. Primary Diagnostic Accuracy in the ICA Cohort

**Figure 2:**
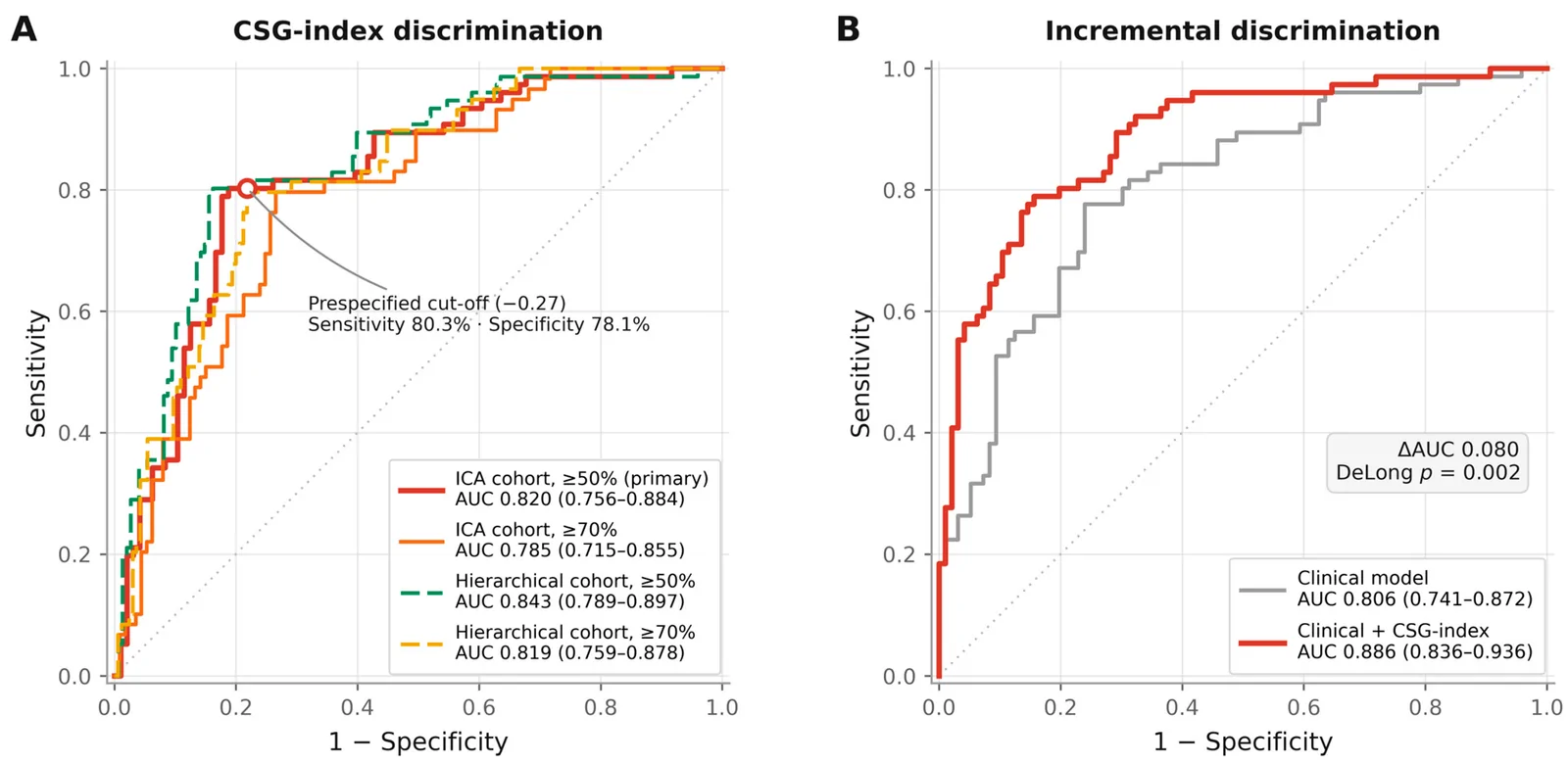
Discrimination of the continuous CSG-index and its incremental value beyond available clinical variables. (**A**) Discriminative performance of the continuous CSG-index for obstructive coronary artery disease (CAD; ≥50% stenosis) and severe CAD (≥70% stenosis) in the primary invasive coronary angiography (ICA) cohort (*n* = 172) and the hierarchical anatomically adjudicated cohort (*n* = 224), which additionally included patients without ICA in whom coronary computed tomography angiography (CCTA) showed no or non-obstructive disease. The open circle indicates the operating point corresponding to the prespecified cut-off (−0.27) on the primary curve. (**B**) Incremental discrimination for obstructive CAD in the ICA cohort. The Clinical model (age, sex, body mass index, current smoking, diabetes mellitus, hypertension, and hyperlipidaemia) was compared with the Clinical + CSG-index model. Areas under the curve (AUCs) are shown with 95% confidence intervals calculated using the DeLong method; ΔAUC denotes the difference between the two models, and the *p*-value was derived using the DeLong test for paired AUCs. The dotted line indicates chance-level performance. Abbreviations: AUC, area under the receiver operating characteristic curve; CAD, coronary artery disease; CCTA, coronary computed tomography angiography; CI, confidence interval; CSG, the artificial intelligence-enabled vectorcardiography-derived index; ICA, invasive coronary angiography.

For ICA-defined obstructive CAD, the continuous CSG-index yielded an AUC of 0.820 (95% CI, 0.756–0.884; Figure 2A). At the prespecified cut-off of −0.27, 61 patients were classified as true positive, 75 as true negative, 21 as false positive, and 15 as false negative. Of the 21 false-positive classifications, 16 occurred in class 0 and 5 in class 1; of the 15 false-negative classifications, 3 occurred in class 2 and 12 in class 3. Sensitivity was 80.3% (95% CI, 70.0–87.7%), specificity 78.1% (68.9–85.2%), positive predictive value 74.4% (64.0–82.6%), negative predictive value 83.3% (74.3–89.6%), and accuracy 79.1% (72.4–84.5%; Table 2). No CSG-index or ICA reference result was missing or indeterminate.

### 3.3. Secondary Anatomical Outcomes

For severe CAD in the ICA cohort, the AUC was 0.785 (95% CI, 0.715–0.855); at the prespecified cut-off, sensitivity was 79.7%, specificity 69.0%, and accuracy 72.7%. In the hierarchical cohort, the AUC was 0.843 (0.789–0.897) for obstructive CAD and 0.819 (0.759–0.878) for severe CAD. The corresponding sensitivities were 80.3% and 79.7%, specificities 81.1% and 74.5%, and accuracies 80.8% and 75.9%, respectively (Table 2; Figure 2A). Among reference-negative patients, a positive CSG-index was observed in 21/96 (21.9%) verified by ICA and 7/52 (13.5%) verified by CCTA alone (*p* = 0.274); median CSG-index values were −0.671 (IQR, −0.943 to −0.308) and −0.831 (IQR, −0.964 to −0.428), respectively (*p* = 0.115).

### 3.4. Incremental Discrimination Beyond Available Clinical Variables

All 172 patients in the ICA cohort had complete data for multivariable modelling. After adjustment for age, sex, body mass index, current smoking, diabetes mellitus, hypertension, and hyperlipidaemia, the CSG-index remained associated with obstructive CAD (OR per 1-SD increase [0.583 index units], 4.07; 95% CI, 2.41–6.87; *p* < 0.001; Table 3). Adding the CSG-index increased the AUC from 0.806 (95% CI, 0.741–0.872) to 0.886 (0.836–0.936; ΔAUC = 0.080; DeLong *p* = 0.002; Figure 2B) and improved model fit (likelihood-ratio χ^2^ = 38.30; *p* < 0.001). After 2000 bootstrap resamples, the optimism-corrected AUCs were 0.776 and 0.862 for the Clinical and Clinical + CSG-index models, respectively, indicating that the improvement in discrimination was retained after optimism correction.

### 3.5. Stenosis-Severity Gradient and Sex-Stratified Analysis

**Figure 3:**
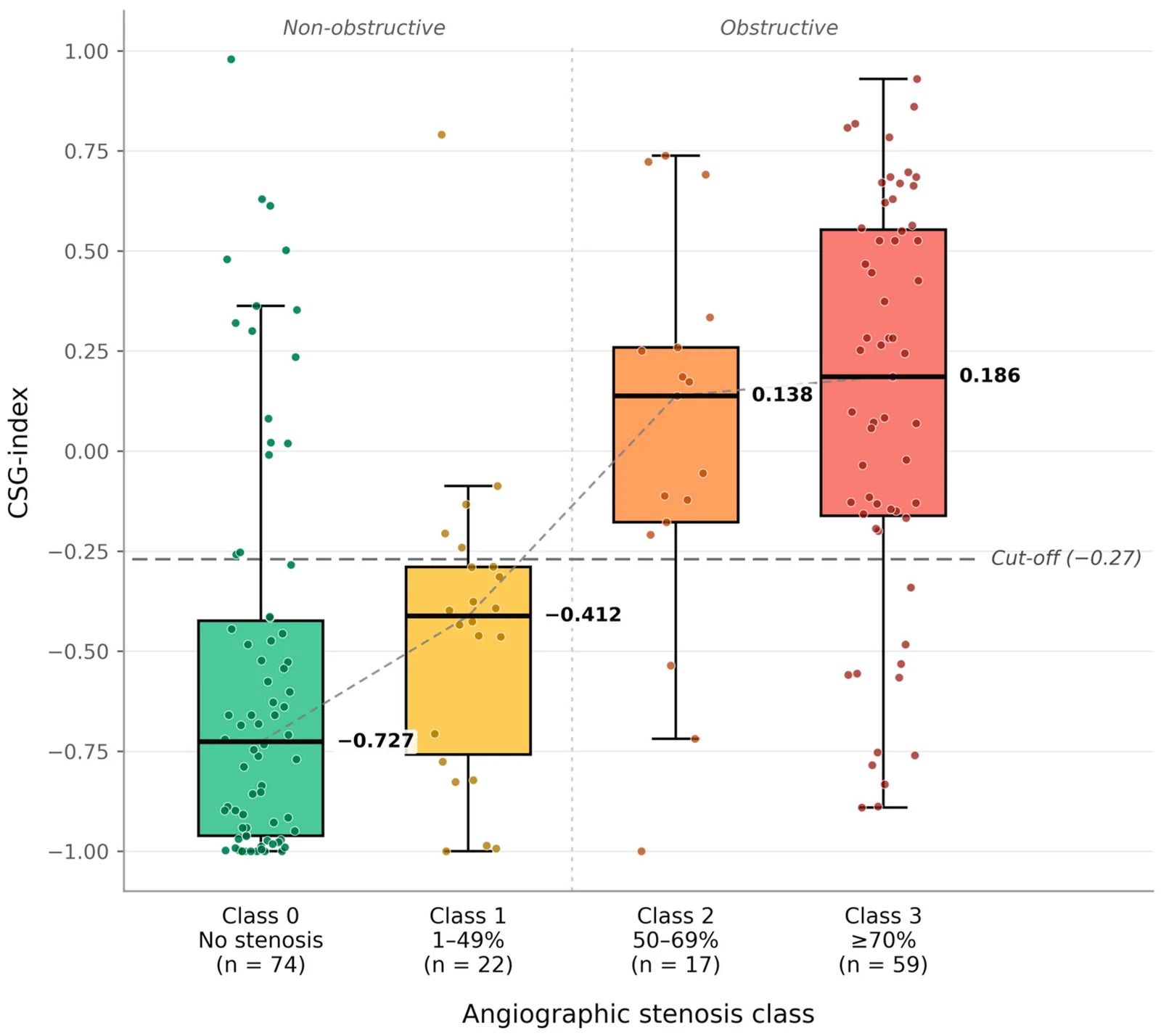
Distribution of the CSG-index across angiographic stenosis classes. Individual points represent patients; boxes indicate the interquartile range, horizontal lines within the boxes indicate medians, and whiskers extend to the most extreme observations within 1.5 times the interquartile range. Median values are labelled and connected by a grey dashed line. The horizontal dashed line indicates the prespecified CSG-index cut-off of −0.27, whereas the vertical dotted line separates non-obstructive (classes 0–1) from obstructive CAD (≥50% stenosis; classes 2–3). Stenosis severity was classified as follows: class 0, no stenosis; class 1, 1–49%; class 2, 50–69%; and class 3, ≥70%. Abbreviations: CAD, coronary artery disease; CSG, the artificial intelligence-enabled vectorcardiography-derived index; ICA, invasive coronary angiography.

Median CSG-index values increased across ICA classes 0–3: −0.727 (IQR, −0.962 to −0.424), −0.412 (−0.759 to −0.289), 0.138 (−0.178 to 0.259), and 0.186 (−0.162 to 0.554), respectively (Figure 3), with a significant ordered trend (Jonckheere–Terpstra *p* < 0.001; Spearman ρ = 0.543, *p* < 0.001). In the proportional-odds model, each one-unit increase in the CSG-index was associated with greater odds of belonging to a higher stenosis class (common OR, 8.07; 95% CI, 4.41–14.77; *p* < 0.001), with no violation of the proportional-odds assumption (Brant *p* = 0.123).

In exploratory sex-stratified analyses, the AUC was 0.845 in women (*n* = 75; 26 events), with a DeLong 95% CI of 0.754–0.936 and a stratified bootstrap 95% CI of 0.749–0.927. In men (*n* = 97; 50 events), the corresponding AUC was 0.833, with a DeLong 95% CI of 0.749–0.918 and a stratified bootstrap 95% CI of 0.747–0.912. The adjusted CSG-index × sex interaction was not significant (*p* = 0.645).

### 3.6. Exploratory Functional-Test Subgroups and Concordance Analysis

Exercise ECG results were available for 83 participants in the ICA cohort, 30 of whom had obstructive CAD. Propensity-score analysis indicated measurable selection into this subgroup. The selection model had an AUC of 0.655, with adequate propensity-score overlap between participants with and without available exercise ECG results.

Stabilised weights ranged from 0.585 to 2.279 and yielded an effective sample size of 158.8. After weighting, all measured covariates were well balanced (maximum absolute SMD, 0.023; Supplementary Table S2). Before weighting, participants who underwent exercise ECG had a lower prevalence of obstructive CAD than those without available exercise ECG results (36.1% vs. 51.7%; *p* = 0.047); after weighting, this difference was attenuated from −15.5 to −5.5 percentage points (95% CI, −19.3 to 8.4).

Exercise ECG sensitivity and specificity were 63.3% and 60.4%, respectively; the corresponding values for the CSG-index were 80.0% and 84.9%. Among the 43 patients in the ICA cohort with available MPS results, 14 had obstructive CAD. MPS sensitivity and specificity were 85.7% and 69.0%, respectively; the corresponding values for the CSG-index were 71.4% and 86.2% (Table 4).

**Figure 4:**
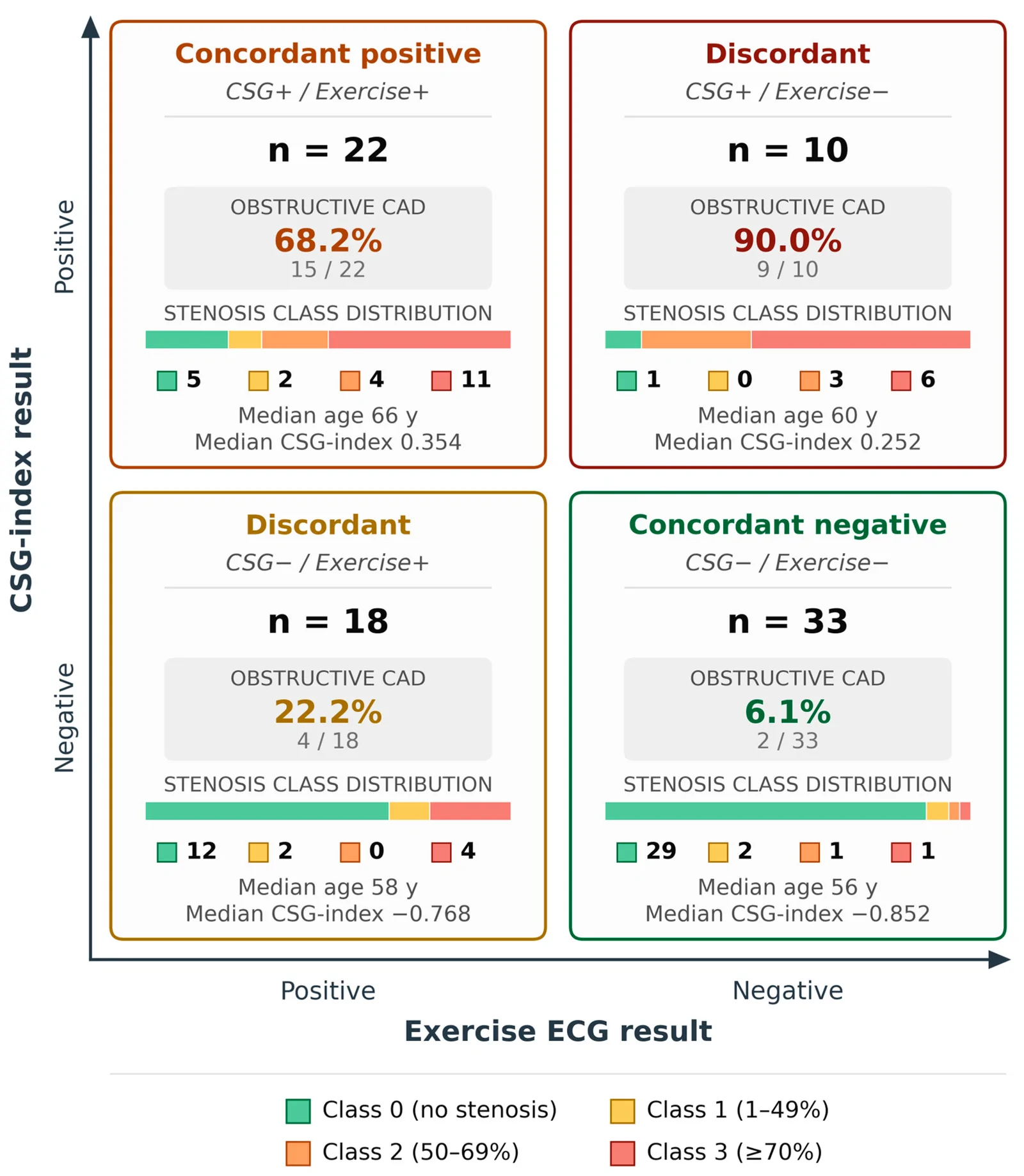
Exploratory concordance and discordance between the CSG-index and exercise ECG in the ICA cohort. Patients were classified into four groups according to the combination of CSG-index results (positive, ≥−0.27) and exercise ECG results (positive or negative, as recorded in the routine clinical report). Each quadrant presents the number of patients, the proportion with obstructive coronary artery disease (≥50% stenosis) on ICA, the distribution of angiographic stenosis classes, and the median age and CSG-index. Quadrant border colour reflects the prevalence of obstructive disease within that quadrant. The two tests were concordant in 55 of 83 patients (66.3%). Stenosis severity was classified as follows: class 0, no stenosis; class 1, 1–49%; class 2, 50–69%; and class 3, ≥70%. Abbreviations: CAD, coronary artery disease; CSG, the artificial intelligence-enabled vectorcardiography-derived index; ECG, electrocardiography; ICA, invasive coronary angiography; y, years.

The CSG-index and exercise ECG classifications were concordant in 55 of 83 patients (66.3%), with distinct distributions of obstructive CAD across the concordant and discordant categories (Figure 4). The CSG-index was positive in 9 of 11 (81.8%) false-negative exercise ECG cases and negative in 14 of 21 (66.7%) false-positive exercise ECG cases; conversely, exercise ECG was positive in 4 of 6 (66.7%) CSG-index false-negative cases and negative in 1 of 8 (12.5%) CSG-index false-positive cases. Among the 28 discordant classifications, the ICA reference classification agreed with the CSG-index in 23 cases and with exercise ECG in 5 cases (exact McNemar *p* < 0.001). In reference-stratified paired comparisons, sensitivity did not differ significantly (9 vs. 4 discordant cases; *p* = 0.267), whereas specificity favoured the CSG-index (14 vs. 1 discordant cases; *p* < 0.001; Figure 4).

## 4. Discussion

In the two-centre, real-world VECTOR-CAD study, the locked CSG-index showed good discrimination for ICA-defined obstructive CAD, with an AUC of 0.820 and balanced sensitivity (80.3%) and specificity (78.1%). Discrimination was maintained in the hierarchical anatomical cohort. Beyond binary classification, CSG-index values increased progressively across stenosis classes, supporting the view that the index does not act solely as a threshold-based classifier but also carries information related to anatomical disease burden. After adjustment for available clinical risk factors, the index remained independently associated with obstructive CAD, and the associated gain in discrimination persisted after bootstrap optimism correction. In the concordance analysis with exercise ECG, the two tests agreed in approximately two-thirds of patients; in exploratory paired comparisons, specificity and overall accuracy favoured the CSG-index. These findings support the CSG-index as a source of complementary diagnostic information in clinically selected patients undergoing evaluation for suspected CAD.

Our findings extend previous evidence on resting three-dimensional electrical assessment. Early cardiogoniometry studies suggested that spatial depolarisation–repolarisation patterns could detect CAD at rest; a seminal study reported 64% sensitivity and 82% specificity against invasive angiography [5], while a subsequent meta-analysis found pooled sensitivity and specificity of 71.7% and 78.8%, respectively, with a summary ROC AUC of 0.81, albeit with substantial heterogeneity (I^2^ > 75%) [11]. Over time, the application of artificial intelligence to standard resting ECG showed that CAD-related patterns not apparent on visual interpretation could be extracted [7,8,9,10,17]. Extending this approach to three-dimensional VCG offered the potential to capture information beyond conventional analysis. Braun et al. reported promising sensitivity for the AI-VCG-derived CSG-index (90.2% in women and 97.2% in men), although these estimates came from an algorithm-development study using nested five-fold cross-validation [12]. Aydar et al. compared AI-VCG with MPS as a functional target rather than anatomical stenosis [13], while Rudland et al. evaluated population-based risk assessment and referral optimisation [14]. Against this background, to our knowledge, this is the first external validation of the locked CSG-index against anatomically confirmed obstructive CAD in a real-world outpatient cardiology cohort.

Performance estimates generated during algorithm development tend to be optimistic because evaluation remains within the same dataset [18,19], and transferring a locked algorithm to a new setting introduces differences in patient spectrum, disease prevalence, and signal acquisition, a phenomenon known as domain shift. A systematic review of diagnostic radiology AI algorithms reported reduced external performance in 81% of the algorithms evaluated [20]. The CSG-index algorithm was originally developed in German patient cohorts [12,14], whereas the present Turkish outpatient population may differ in demographic and cardiovascular risk profiles, body habitus, referral pathways, disease prevalence, and recording conditions. Our more modest sensitivity relative to Braun et al. should be interpreted in this context. However, the two studies are not directly comparable, because the positive target in their study was angiographic CAD involving one, two, or three vessels without a quantitative stenosis threshold [12]. Because our performance estimates were obtained in an external target population, they reflect algorithm performance after transfer to a new setting, although the contribution of domain shift cannot be separated from differences in study design, patient selection, and reference-standard definitions.

Discrimination of the index was similar across sex strata, with AUCs of 0.845 in women and 0.833 in men. The DeLong and bootstrap confidence intervals were similar, and no significant CSG-index × sex interaction was detected (*p* = 0.645; Table 2). These findings suggest that the index performed similarly in both sexes. However, this analysis was exploratory and included only 26 obstructive events among women; therefore, the absence of a significant interaction should not be interpreted as evidence of equivalent performance between the sexes.

The hierarchical cohort was designed as a supportive sensitivity analysis of mixed outpatient diagnostic pathways rather than as a fully representative outpatient cohort. Restricting analysis to invasively investigated patients may underrepresent verified negatives and characterise negative performance only in a higher-risk subgroup, whereas combining ICA and CCTA may introduce differential verification bias [15,16]. Because CCTA has high sensitivity but more modest specificity for obstructive CAD, a negative result provides stronger evidence for disease exclusion than a positive result does for disease presence [4,21,22]. We therefore adopted a pragmatic strategy in which ICA remained the sole means of confirming reference-positive disease, while CCTA was used only to define reference-negative status in patients without ICA. After adding 52 CCTA-only reference-negative cases, discrimination remained similar (AUC 0.843 vs. 0.820; Table 2). Because the same 76 reference-positive cases were included in both cohorts, sensitivity was unchanged; differences in AUC and specificity reflected the broader negative-case spectrum, whereas higher NPV and accuracy also reflected the lower disease prevalence. These estimates therefore do not indicate an intrinsic improvement in index performance. CCTA-only negatives had numerically lower median CSG-index values (−0.831 vs. −0.671) but positivity rates that did not differ significantly (13.5% vs. 21.9%); however, this underpowered post hoc comparison cannot exclude index-related referral or confounding by clinical risk.

An important finding was the ordered increase in CSG-index across stenosis classes. Its moderate correlation with stenosis class (ρ = 0.543), significant monotonic trend, and common OR of 8.07 per one-unit increase suggest that the index does not behave merely as a binary classifier but captures information related to anatomical disease burden. Median values increased from class 0 (−0.727) to class 1 (−0.412) and class 2 (0.138), but changed little in class 3 (0.186), suggesting sharper discrimination between non-obstructive and obstructive disease than grading within the obstructive range (Figure 3). One plausible interpretation is that the CSG-index reflects the myocardial electrical consequences of ischaemic burden rather than the percentage of luminal stenosis itself, consistent with the imperfect concordance between anatomical severity and haemodynamic significance [23,24]. However, without mechanistic imaging, quantitative perfusion, or invasive physiological assessment, the basis of this gradient and any causal relationship between epicardial stenosis and the CSG-index cannot be established.

The CSG-index remained strongly and independently associated with obstructive CAD after adjustment for age, sex, body mass index, current smoking, diabetes mellitus, hypertension, and hyperlipidaemia (OR per 1-SD increase, 4.07; 95% CI, 2.41–6.87; *p* < 0.001). Adding the index to the Clinical model increased the AUC from 0.806 to 0.886 (ΔAUC = 0.080; DeLong *p* = 0.002) and significantly improved model fit (likelihood-ratio test *p* < 0.001). After 2000 bootstrap resamples, optimism-corrected AUCs were 0.776 for the Clinical model and 0.862 for the Clinical + CSG-index model, indicating that the gain in discrimination was largely retained (Table 3 and Figure 2B). These findings indicate that the CSG-index provided incremental discrimination beyond the available demographic and clinical risk variables, consistent with prior evidence on AI-enabled electrical analysis [10]. The models assessed incremental discrimination rather than individual risk prediction or clinical decision-making; therefore, no conclusions can be drawn regarding calibration, patient-level reclassification, or clinical net benefit.

In the exploratory functional-test subgroups, exercise ECG (*n* = 83; 30 with obstructive CAD) showed 63.3% sensitivity, 60.4% specificity, and 61.4% accuracy, compared with 80.0%, 84.9%, 83.1%, and an AUC of 0.854 for the CSG-index in the same patients (Table 4). The close agreement of our exercise ECG findings with the meta-analytic estimates of 0.66 for sensitivity and 0.61 for specificity suggests that exercise ECG performed as expected in this subgroup, although test selection was clinically determined and not standardised [25]. The exercise ECG subgroup was younger and had a lower crude prevalence of obstructive CAD than the remainder of the ICA cohort. After propensity-score weighting, the measured clinical characteristics were well balanced and the prevalence difference was substantially attenuated. However, selection related to unmeasured factors, including symptoms and clinical indications for testing, cannot be excluded. Findings from this subgroup should therefore remain exploratory.

In the MPS subgroup (*n* = 43; 14 with obstructive CAD), the CSG-index yielded an AUC of 0.833; MPS had higher sensitivity, whereas the CSG-index had higher specificity (Table 4). No paired statistical comparison was performed because of the small subgroup and limited number of events. MPS assesses myocardial perfusion abnormalities, whereas the reference endpoint in our study was anatomical stenosis ≥50%; the relationship between anatomical stenosis and functional ischaemia is not always consistent [22,26]. Using MPS as the reference test, Aydar et al. reported incomplete agreement with AI-VCG, characterised by high specificity despite low sensitivity, and positioned the method as a potential rule-out tool [13].

In the exploratory concordance analysis, exercise ECG and the CSG-index were concordant in 66.3% of patients. Among the 10 CSG-positive/exercise ECG-negative patients, 9 (90.0%) had obstructive CAD, whereas 4 of the 18 (22.2%) CSG-negative/exercise ECG-positive patients had obstructive CAD (Figure 4). Among the 28 discordant classifications, the ICA reference classification agreed with the CSG-index in 23 cases and with exercise ECG in 5. Paired comparisons favoured the CSG-index for specificity, whereas sensitivity did not differ significantly, indicating that its higher overall accuracy was driven mainly by fewer false-positive results. This discordance may reflect differences in the information captured by the tests: exercise ECG detects workload-dependent ST-segment changes related to myocardial oxygen supply–demand imbalance, whereas resting AI-VCG analyses spatial depolarisation–repolarisation characteristics without stress induction [5,12]. The two tests may therefore not be expected to agree completely. However, the analysis was based on a small, clinically selected subgroup without standardised testing indications and was not designed as a prospective head-to-head comparison. It therefore does not demonstrate that Cardisiography is clinically superior to exercise ECG or can replace or reduce the use of exercise ECG, MPS, CCTA, or ICA. Whether these differing classification patterns provide useful complementary information remains a hypothesis that requires prospective evaluation at a prespecified point in the diagnostic pathway.

The practical potential of AI-VCG lies in an approximately four-minute resting recording with low operator dependence and no need for radiation, contrast, or stress induction. These features raise the hypothesis that, following clinical assessment, the CSG-index could inform decisions before selected patients are referred for more resource-intensive tests with potential adverse effects. Current guidelines recommend selecting tests according to clinical likelihood, exercise capacity, ECG interpretability, patient characteristics, local expertise, and whether the clinical question is anatomical or functional [4,27]. The −0.27 reference cut-off yields a balanced rather than a rule-out or rule-in profile. At this cut-off, a quarter of false-positive classifications occurred in patients with non-obstructive plaque, whereas false-negative classifications were distributed in proportion to disease severity. False-positive results could therefore prompt unnecessary downstream testing, whereas false-negative results could delay the diagnosis of obstructive CAD, including severe disease; because predictive values depend on disease prevalence, the relative weight of these errors would differ across clinical settings. The balanced performance of the CSG-index and its incremental discrimination beyond the available clinical variables are consistent with this potential role, which remains a hypothesis for prospective evaluation. Accordingly, AI-VCG should not be used as a standalone rule-in or rule-out test or as a substitute for CCTA, functional testing, or ICA [4,21,27]. Future studies should prospectively validate AI-VCG in multicentre consecutive cohorts across different disease prevalences and referral patterns, extending evaluation beyond diagnostic accuracy to downstream testing, time to diagnosis, cost-effectiveness, and clinical outcomes.

### Strengths and Limitations

The principal strength of this study is the evaluation of a locked CSG-index in a two-centre, real-world cohort without retraining, recalibration, or threshold optimisation. ICA was retained as the primary reference standard, and a prespecified threshold was used. Continuous and threshold-based performance was complemented by analyses of severe CAD, the stenosis-severity gradient, the hierarchical anatomical cohort, and incremental discrimination beyond the available clinical variables. Additional strengths included complete index-test, reference-standard, and modelling data in the primary cohort, bootstrap optimism correction, full confusion-matrix reporting, the absence of indeterminate results, and comparison of the functional tests against the same ICA reference, including paired error patterns with exercise ECG.

This study also has several limitations. Its retrospective design and clinically determined downstream testing introduce potential selection, referral, spectrum, and partial verification biases because inclusion in the primary ICA cohort depended on routine clinical referral rather than a study-defined pathway. Because Cardisiography results were available when anatomical testing was selected, an influence on individual referral decisions cannot be excluded, although no study protocol or prespecified CSG-index criterion determined referral. Any resulting bias in diagnostic performance cannot be quantified from these retrospective data.

The hierarchical analysis added reference-negative cases verified by CCTA alone and broadened the negative-case spectrum but did not eliminate differential verification bias. Reference-standard readers were not blinded to Cardisiography findings or other clinical information, ICA stenosis was estimated visually without quantitative coronary angiography, and no central or independent duplicate adjudication was performed. Awareness of the CSG-index may therefore have introduced review bias, potentially increasing agreement with the reference standard, particularly for lesions near the stenosis thresholds.

The clinical model did not include symptom characteristics, resting ECG findings, or an established pre-test likelihood estimate, as these were not systematically recorded; the incremental discrimination of the CSG-index should therefore be interpreted relative to the available variables rather than to a complete contemporary clinical assessment.

The exercise ECG and MPS analyses were exploratory available-case comparisons in small, clinically selected subgroups without standardised indications or a prospective head-to-head design. Thus, differences in diagnostic metrics or paired classifications do not establish clinical superiority, interchangeability, or a reduced need for established anatomical or functional tests. Net benefit, cost-effectiveness, downstream testing decisions, and patient outcomes were not assessed. Sample size and event counts were limited, particularly in the functional-test and sex-stratified analyses.

The system’s saliency maps, feature-attribution values, intermediate model representations, and model weights were proprietary and were not available to the investigators. Therefore, patient-level explainability analyses, including analyses of false-positive and false-negative classifications, could not be performed in this study.

Finally, the location of both centres within the same geographic region may restrict generalisability to populations with different demographic, clinical, and recording characteristics.

## 5. Conclusions

In this two-centre, real-world validation study, the locked CSG-index demonstrated balanced diagnostic performance for ICA-defined obstructive CAD at the prespecified threshold, and its discrimination was maintained for severe CAD and in the hierarchical anatomical cohort. The index provided graded information associated with stenosis severity and incremental discrimination beyond the available demographic and clinical risk variables. It also maintained discrimination in the exploratory functional-test subgroups and showed limited concordance with exercise ECG, with fewer false-positive classifications. However, these exploratory findings should not be interpreted as evidence of clinical superiority or as support for replacing established anatomical or functional tests. These findings support further investigation of the CSG-index as a complementary resting test in patients undergoing evaluation for suspected CAD. Its role within the diagnostic pathway and its clinical utility should be determined through prospective multicentre validation and impact studies.

## Figures and Tables

**Table 1:** Baseline characteristics of the invasive coronary angiography (ICA) cohort, stratified by obstructive coronary artery disease (CAD).

Characteristic	Overall (*n* = 172)	Non-Obstructive (*n* = 96) Class 0–1	Obstructive (*n* = 76) Class 2–3	*p* ^1^
Age, years	60.0 (53.0–68.0)	57.0 (51.0–65.0)	63.0 (58.0–70.3)	<0.001
Female sex, *n* (%)	75 (43.6)	49 (51.0)	26 (34.2)	0.031
Body mass index, kg/m^2^	29.1 (26.1–32.0)	28.9 (25.7–32.0)	29.2 (26.7–32.7)	0.379
Diabetes mellitus, *n* (%)	58 (33.7)	25 (26.0)	33 (43.4)	0.023
Hypertension, *n* (%)	105 (61.0)	42 (43.8)	63 (82.9)	<0.001
Hyperlipidaemia, *n* (%)	56 (32.6)	29 (30.2)	27 (35.5)	0.514
Current smoking, *n* (%)	47 (27.3)	29 (30.2)	18 (23.7)	0.391
CSG-index	−0.3 (−0.8 to 0.3)	−0.7 (−0.9 to −0.3)	0.2 (−0.2 to 0.5)	<0.001
Positive CSG-index, *n* (%)	82 (47.7)	21 (21.9)	61 (80.3)	<0.001
Exercise ECG performed, *n* (%)	83 (48.3)	53 (55.2)	30 (39.5)	—
Positive exercise ECG, *n*/tested (%)	40/83 (48.2)	21/53 (39.6)	19/30 (63.3)	—
MPS performed, *n* (%)	43 (25.0)	29 (30.2)	14 (18.4)	—
Positive MPS, *n*/tested (%)	21/43 (48.8)	9/29 (31.0)	12/14 (85.7)	—

Continuous variables are presented as median (interquartile range), and categorical variables as *n* (%). Obstructive CAD was defined as ≥50% stenosis (class 2–3) on invasive coronary angiography. A positive index test was defined by the prespecified CSG-index cut-off of −0.27. Positive exercise ECG and MPS results are expressed as *n* positive/*n* tested (%). ^1^ *p*-values were derived using the Mann–Whitney U test for continuous variables and Fisher’s exact test for categorical variables; no *p*-value is reported for test-availability rows (—). AI-VCG, artificial intelligence-enabled resting vectorcardiography; CSG, the AI-VCG-derived index; ECG, electrocardiography; ICA, invasive coronary angiography; MPS, myocardial perfusion scintigraphy.

**Table 2:** Diagnostic performance and receiver operating characteristic (ROC) discrimination of the CSG-index at the prespecified cut-off (−0.27).

Analysis	*n*	Events ^1^	Sensitivity	Specificity	PPV	NPV	Accuracy	AUC ^2^
ICA cohort, ≥50% (primary)	172	76	80.3 (70.0–87.7)	78.1 (68.9–85.2)	74.4 (64.0–82.6)	83.3 (74.3–89.6)	79.1 (72.4–84.5)	0.820 (0.756–0.884)
ICA cohort, ≥70% (severe)	172	59	79.7 (67.7–88.0)	69.0 (60.0–76.8)	57.3 (46.5–67.5)	86.7 (78.1–92.2)	72.7 (65.6–78.8)	0.785 (0.715–0.855)
Hierarchical cohort, ≥50%	224	76	80.3 (70.0–87.7)	81.1 (74.0–86.6)	68.5 (58.3–77.2)	88.9 (82.5–93.1)	80.8 (75.1–85.4)	0.843 (0.789–0.897)
Hierarchical cohort, ≥70%	224	59	79.7 (67.7–88.0)	74.5 (67.4–80.6)	52.8 (42.5–62.8)	91.1 (85.1–94.8)	75.9 (69.9–81.0)	0.819 (0.759–0.878)
Female, ≥50% (exploratory) ^3^	75	26	84.6 (66.5–93.8)	73.5 (59.7–83.8)	62.9 (46.3–76.8)	90.0 (76.9–96.0)	77.3 (66.7–85.3)	0.845 (0.754–0.936)
Male, ≥50% (exploratory) ^3^	97	50	78.0 (64.8–87.2)	83.0 (69.9–91.1)	83.0 (69.9–91.1)	78.0 (64.8–87.2)	80.4 (71.4–87.1)	0.833 (0.749–0.918)

All performance metrics are presented as percentages with 95% confidence intervals. Diagnostic performance was assessed using the prespecified CSG-index cut-off of −0.27. The hierarchical cohort comprises the ICA cohort and patients without ICA whose CCTA demonstrated class 0–1 disease; the latter contributed only as reference-negative cases. ^1^ Events denote the number of patients with the reference-standard target condition at the corresponding threshold. ^2^ Confidence intervals were calculated using the Wilson score method for sensitivity, specificity, PPV, NPV, and accuracy and the DeLong method for AUC. ^3^ Sex-stratified analyses are exploratory. In post hoc analyses, the 2000-resample stratified percentile bootstrap 95% CIs for AUC were 0.749–0.927 in women and 0.747–0.912 in men. AUC, area under the ROC curve; CCTA, coronary computed tomography angiography; CSG, artificial intelligence-enabled vectorcardiography-derived index; ICA, invasive coronary angiography; NPV, negative predictive value; PPV, positive predictive value.

**Table 3:** Multivariable logistic regression models for obstructive coronary artery disease (ICA cohort, complete-case analysis, *n* = 172).

Variable	Clinical Model, OR (95% CI)	Clinical + CSG-Index Model, OR (95% CI)
Age, per year	1.06 (1.02–1.10)	1.03 (0.98–1.08)
Male sex	3.98 (1.68–9.41)	7.03 (2.46–20.12)
Body mass index, per kg/m^2^	1.02 (0.95–1.10)	1.07 (0.98–1.16)
Diabetes mellitus	1.46 (0.66–3.23)	1.04 (0.41–2.63)
Hypertension	6.53 (2.83–15.06)	5.18 (1.98–13.56)
Hyperlipidaemia	0.93 (0.42–2.07)	1.18 (0.47–2.99)
Current smoking	0.89 (0.35–2.22)	0.67 (0.24–1.88)
CSG-index, per 1-SD	—	4.07 (2.41–6.87)
Model AUC	0.806 (0.741–0.872); optimism-corrected 0.776	0.886 (0.836–0.936); optimism-corrected 0.862
DeLong *p* versus clinical model	—	0.002
Likelihood-ratio test *p*	—	<0.001

Odds ratios (ORs) with 95% confidence intervals (CIs) were derived from logistic regression for obstructive CAD (≥50% stenosis). Both models were prespecified; the Clinical model comprised age, sex, body mass index, diabetes mellitus, hypertension, hyperlipidaemia, and current smoking. The CSG-index was entered per 1-standard-deviation (SD) increase (1 SD = 0.583 index units). Optimism-corrected AUCs were estimated using 2000 bootstrap resamples. The DeLong test compares the AUCs of the two models, and the likelihood-ratio test compares their model fit. A thin rule separates variable-level estimates from model-level performance indices. AUC, area under the receiver operating characteristic curve; CAD, coronary artery disease; CSG, artificial intelligence-enabled vectorcardiography-derived index; ICA, invasive coronary angiography.

**Table 4:** Exploratory functional-comparator analyses in tested subgroups of the ICA cohort.

Test/Subgroup	*n*	Events ^1^	Sensitivity	Specificity	PPV	NPV	Accuracy	AUC ^2^
Exercise ECG (binary)	83	30	63.3 (45.5–78.1)	60.4 (46.9–72.4)	47.5 (32.9–62.5)	74.4 (59.8–85.1)	61.4 (50.7–71.2)	—
CSG-index within exercise subgroup	83	30	80.0 (62.7–90.5)	84.9 (72.9–92.1)	75.0 (57.9–86.7)	88.2 (76.6–94.5)	83.1 (73.7–89.7)	0.854 (0.772–0.936)
MPS (binary)	43	14	85.7 (60.1–96.0)	69.0 (50.8–82.7)	57.1 (36.5–75.5)	90.9 (72.2–97.5)	74.4 (59.8–85.1)	—
CSG-index within MPS subgroup	43	14	71.4 (45.4–88.3)	86.2 (69.4–94.5)	71.4 (45.4–88.3)	86.2 (69.4–94.5)	81.4 (67.4–90.3)	0.833 (0.702–0.963)

All performance metrics are presented as percentages with Wilson 95% confidence intervals. ^1^ Events denote the number of patients with obstructive CAD (≥50% stenosis) within each tested subgroup. ^2^ For binary functional tests, AUC is not defined for a single operating point and is therefore not reported (—); for the continuous CSG-index, AUC was calculated from CSG-index values within the corresponding subgroup. Functional tests were performed on a clinically selected basis and were not included in the primary analysis; these results are hypothesis-generating and should be interpreted with caution given the small subgroup sizes and wide confidence intervals. AUC, area under the receiver operating characteristic curve; CAD, coronary artery disease; CSG, the artificial intelligence-enabled vectorcardiography-derived index; ECG, electrocardiography; ICA, invasive coronary angiography; MPS, myocardial perfusion scintigraphy; NPV, negative predictive value; PPV, positive predictive value.

## Data Availability

The datasets analysed during the current study are not publicly available due to institutional and ethical restrictions but may be available from the corresponding author upon reasonable request and subject to appropriate institutional approval.

## Supplementary Materials

The following supporting information can be downloaded at: https://www.mdpi.com/article/10.3390/diagnostics16203266/s1, Table S1: Baseline characteristics of the full hierarchical anatomically adjudicated cohort (*n* = 224), stratified by obstructive coronary artery disease (CAD); Table S2: Clinical characteristics and propensity-score balance among participants in the primary invasive coronary angiography (ICA) cohort with and without available exercise electrocardiography (ECG) results; Checklist S1: STARD 2015 checklist.
