# Supplementary material for: Real-World Validation of the AI-Enabled Vectorcardiographic CSG-Index for Obstructive Coronary Artery Disease: The VECTOR-CAD Study
Source: Diagnostics (Basel). 2026 Oct 9;16(20):3266. doi: 10.3390/diagnostics16203266 (PMC13703597; doi:10.3390/diagnostics16203266)
Supplement: Supplementary file 1 [file diagnostics-16-03266-s001.zip › STARD Check List Vector CAD.pdf]

Checklist S1. STARD 2015 checklist

Manuscript: Real-World Validation of the AI-Enabled Vectorcardiographic CSG-Index for Obstructive Coronary Artery Disease: The VECTOR-CAD Study

Page and line numbers refer to the revised manuscript with continuous line numbering.

Section & Topic	No	Item	Reported on page #
**TITLE OR ABSTRACT**			
	**1**	Identification as a study of diagnostic accuracy using at least one measure of accuracy (such as sensitivity, specificity, predictive values, or AUC)	p. 1, lines 15–16, 24–26 (Abstract)
**ABSTRACT**			
	**2**	Structured summary of study design, methods, results, and conclusions (for specific guidance, see STARD for Abstracts)	p. 1, lines 11–33 (Abstract)
**INTRODUCTION**			
	**3**	Scientific and clinical background, including the intended use and clinical role of the index test	pp. 1–2, lines 38–86 (Section 1)
	**4**	Study objectives and hypotheses	pp. 2–3, lines 87–100 (Section 1)
**METHODS**			
Study design	**5**	Whether data collection was planned before the index test and reference standard were performed (prospective study) or after (retrospective study)	p. 3, lines 103–104 (Section 2.1)
Participants	**6**	Eligibility criteria	p. 3, lines 112–117 (Section 2.1)
	**7**	On what basis potentially eligible participants were identified (such as symptoms, results from previous tests, inclusion in registry)	p. 3, lines 103–105, 112–113 (Section 2.1)
	**8**	Where and when potentially eligible participants were identified (setting, location and dates)	p. 3, lines 103–107 (Section 2.1)
	**9**	Whether participants formed a consecutive, random or convenience series	p. 3, lines 118–119; p. 6, lines 217–218[a]
Test methods	**10a**	Index test, in sufficient detail to allow replication	pp. 5–6, lines 139–193 (Section 2.2)
	**10b**	Reference standard, in sufficient detail to allow replication	p. 6, lines 194–211 (Section 2.3)
	**11**	Rationale for choosing the reference standard (if alternatives exist)	p. 2, lines 75–86; p. 6, lines 204–211; p. 17, lines 520–529
	**12a**	Definition of and rationale for test positivity cut-offs or result categories of the index test, distinguishing pre-specified from exploratory	p. 6, lines 185–190 (Section 2.2)
	**12b**	Definition of and rationale for test positivity cut-offs or result categories of the reference standard, distinguishing pre-specified from exploratory	p. 6, lines 196–202 (Section 2.3)
	**13a**	Whether clinical information and reference standard results were available to the performers/readers of the index test	p. 3, lines 107–111; p. 5, lines 153–154[b]
	**13b**	Whether clinical information and index test results were available to the assessors of the reference standard	p. 6, lines 212–217; p. 19, lines 644–650
Analysis	**14**	Methods for estimating or comparing measures of diagnostic accuracy	pp. 7–8, lines 241–282 (Section 2.5)
	**15**	How indeterminate index test or reference standard results were handled	p. 6, lines 208–211; p. 8, lines 283–285; p. 9, lines 332–333
	**16**	How missing data on the index test and reference standard were handled	p. 8, lines 283–286; p. 9, lines 332–333
	**17**	Any analyses of variability in diagnostic accuracy, distinguishing pre-specified from exploratory	pp. 7–8, lines 251–288; pp. 10–14, lines 352–455
	**18**	Intended sample size and how it was determined	p. 3, lines 117–119 (Section 2.1)

CC BY SA

**RESULTS**			
Participants	**19**	Flow of participants, using a diagram	p. 4 (Figure 1); p. 5, lines 124–138; p. 8, lines 301–302
	**20**	Baseline demographic and clinical characteristics of participants	p. 8, lines 300–313; Table 1 (pp. 8–9); Table S1
	**21a**	Distribution of severity of disease in those with the target condition	p. 8, lines 303–305; p. 12, lines 396–403; Figures 1 and 3
	**21b**	Distribution of alternative diagnoses in those without the target condition	Not reported[c]
	**22**	Time interval and any clinical interventions between index test and reference standard	p. 3, lines 107–109, 116–117[d]
Test results	**23**	Cross tabulation of the index test results (or their distribution) by the results of the reference standard	p. 9, lines 325–329 (Section 3.2)
	**24**	Estimates of diagnostic accuracy and their precision (such as 95% confidence intervals)	p. 9, lines 325–333; pp. 10–14, lines 352–455; Tables 2–4
	**25**	Any adverse events from performing the index test or the reference standard	Not reported[e]
**DISCUSSION**			
	**26**	Study limitations, including sources of potential bias, statistical uncertainty, and generalisability	p. 19, lines 635–668 (Section 4.1)
	**27**	Implications for practice, including the intended use and clinical role of the index test	pp. 18–19, lines 603–623; p. 20, lines 670–682
**OTHER INFORMATION**			
	**28**	Registration number and name of registry	Not registered[f]
	**29**	Where the full study protocol can be accessed	Available on request[g]
	**30**	Sources of funding and other support; role of funders	p. 20, lines 694–695; pp. 20–21, lines 712–719

Notes

[a] All patients meeting the eligibility criteria during the study period were included (consecutive series); no sampling was performed.

[b] Recordings were acquired during routine clinical care, when clinical information was available to the treating staff; the CSG-index was generated automatically by the cloud-based algorithm without human interpretation. Because Cardisiography was performed before the anatomical reference examination, reference-standard results were not available.

[c] Alternative diagnoses in patients without obstructive CAD were not systematically recorded; the distribution of non-obstructive disease (classes 0–1) is reported on p. 8, lines 303–307, and in Figure 1 (p. 4).

[d] Cardisiography preceded the anatomical reference examination by ≤30 days. No coronary revascularisation was performed between the index test and the reference examination.

[e] Adverse events were not systematically recorded in this retrospective study; Cardisiography is a non-invasive resting recording.

[f] Not registered in ClinicalTrials.gov or any other registry (retrospective diagnostic-accuracy study).

[g] The study protocol is available from the corresponding author upon reasonable request.

STARD 2015

AIM

STARD stands for "Standards for Reporting Diagnostic accuracy studies". This list of items was developed to contribute to the completeness and transparency of reporting of diagnostic accuracy studies. Authors can use the list to write informative study reports. Editors and peer-reviewers can use it to evaluate whether the information has been included in manuscripts submitted for publication.

EXPLANATION

A **diagnostic accuracy study** evaluates the ability of one or more medical tests to correctly classify study participants as having a **target condition.** This can be a disease, a disease stage, response or benefit from therapy, or an event or condition in the future. A medical test can be an imaging procedure, a laboratory test, elements from history and physical examination, a combination of these, or any other method for collecting information about the current health status of a patient.

The test whose accuracy is evaluated is called **index test.** A study can evaluate the accuracy of one or more index tests. Evaluating the ability of a medical test to correctly classify patients is typically done by comparing the distribution of the index test results with those of the **reference standard**. The reference standard is the best available method for establishing the presence or absence of the target condition. An accuracy study can rely on one or more reference standards.

If test results are categorized as either positive or negative, the cross tabulation of the index test results against those of the reference standard can be used to estimate the **sensitivity** of the index test (the proportion of participants *with* the target condition who have a positive index test), and its **specificity** (the proportion *without* the target condition who have a negative index test). From this cross tabulation (sometimes referred to as the contingency or "2x2" table), several other accuracy statistics can be estimated, such as the positive and negative **predictive values** of the test. Confidence intervals around estimates of accuracy can then be calculated to quantify the statistical **precision** of the measurements.

If the index test results can take more than two values, categorization of test results as positive or negative requires a **test positivity cut-off**. When multiple such cut-offs can be defined, authors can report a receiver operating characteristic (ROC) curve which graphically represents the combination of sensitivity and specificity for each possible test positivity cut-off. The **area under the ROC curve** informs in a single numerical value about the overall diagnostic accuracy of the index test.

The **intended use** of a medical test can be diagnosis, screening, staging, monitoring, surveillance, prediction or prognosis. The **clinical role** of a test explains its position relative to existing tests in the clinical pathway. A replacement test, for example, replaces an existing test. A triage test is used before an existing test; an add-on test is used after an existing test.

Besides diagnostic accuracy, several other outcomes and statistics may be relevant in the evaluation of medical tests. Medical tests can also be used to classify patients for purposes other than diagnosis, such as staging or prognosis. The STARD list was not explicitly developed for these other outcomes, statistics, and study types, although most STARD items would still apply.

DEVELOPMENT

This STARD list was released in 2015. The 30 items were identified by an international expert group of methodologists, researchers, and editors. The guiding principle in the development of STARD was to select items that, when reported, would help readers to judge the potential for bias in the study, to appraise the applicability of the study findings and the validity of conclusions and recommendations. The list represents an update of the first version, which was published in 2003.

More information can be found on http://www.equator-network.org/reporting-guidelines/stard.
