# Supplementary material for: Real-World Validation of the AI-Enabled Vectorcardiographic CSG-Index for Obstructive Coronary Artery Disease: The VECTOR-CAD Study
Source: Diagnostics (Basel). 2026 Oct 9;16(20):3266. doi: 10.3390/diagnostics16203266 (PMC13703597; doi:10.3390/diagnostics16203266)
Supplement: Supplementary file 1 [file diagnostics-16-03266-s001.zip › Supplementary Table S1.pdf]

Supplementary Table S1. Baseline characteristics of the full hierarchical anatomically adjudicated cohort (n = 224), stratified by obstructive coronary artery disease (CAD).

Characteristic	Overall (n = 224)	Non-Obstructive (n = 148)<br>Class 0-1	Obstructive (n = 76)<br>Class 2-3	p [1]
Age, years	58.0 (50.0–66.0)	55.0 (47.0–62.0)	63.0 (58.0–70.3)	<0.001
Female sex, n (%)	93 (42)	67 (45)	26 (34)	0.118
Body mass index, kg/m²	28.4 (25.8–31.5)	27.7 (25.4–31.2)	29.2 (26.7–32.7)	0.040
Diabetes mellitus, n (%)	66 (29)	33 (22)	33 (43)	0.002
Hypertension, n (%)	125 (56)	62 (42)	63 (83)	<0.001
Hyperlipidaemia, n (%)	62 (28)	35 (24)	27 (36)	0.082
Current smoking, n (%)	62 (28)	44 (30)	18 (24)	0.430
CSG-index	-0.5 (-0.9 to 0.1)	-0.7 (-1.0 to -0.4)	0.2 (-0.2 to 0.5)	<0.001
Positive CSG-index, n (%)	89 (40)	28 (19)	61 (80)	<0.001
Exercise ECG performed, n (%)	102 (46)	72	30	—
Positive exercise ECG, n/tested (%)	45/102 (44)	26/72 (36)	19/30 (63)	—
MPS performed, n (%)	46 (21)	32	14	—
Positive MPS, n/tested (%)	21/46 (46)	9/32 (28)	12/14 (86)	—

The hierarchical cohort comprises the invasive coronary angiography (ICA) cohort together with the non-obstructive coronary computed tomography angiography (CCTA) extension. Positive obstructive CAD (≥50% stenosis) was defined solely by ICA; CCTA contributed only negative adjudication (class 0–1) in patients without invasive angiography. Continuous variables are presented as median (interquartile range) and categorical variables as n (%). Positive exercise ECG and positive MPS are expressed as n positive / n tested (%). [1] p-values were derived from the Mann–Whitney U test for continuous variables and the Fisher exact test for categorical variables; no p-value is reported for test-availability rows (—). CSG, the AI-VCG-derived index; ECG, electrocardiography; MPS, myocardial perfusion scintigraphy.
