# Supplementary material for: Real-World Validation of the AI-Enabled Vectorcardiographic CSG-Index for Obstructive Coronary Artery Disease: The VECTOR-CAD Study
Source: Diagnostics (Basel). 2026 Oct 9;16(20):3266. doi: 10.3390/diagnostics16203266 (PMC13703597; doi:10.3390/diagnostics16203266)
Supplement: Supplementary file 1 [file diagnostics-16-03266-s001.zip › Supplementary Table S2.pdf]

Table S2. Clinical characteristics and propensity-score balance among participants in the primary invasive coronary angiography (ICA) cohort with and without available exercise electrocardiography (ECG) results.

(a) Variables included in the propensity model

	Exercise ECG			Absolute SMD	
**Characteristic**	**Available (n = 83)**	**Not available (n = 89)**	***p*** [1]	**Unweighted**	**Weighted** [2]
Age, years	60.0 (51.0–65.0)	61.0 (54.0–70.0)	0.028	0.378	0.010
Female sex, n (%)	39 (47.0)	36 (40.4)	0.443	0.131	0.021
Body mass index, kg/m²	28.9 (25.4–32.0)	29.1 (27.3–32.4)	0.120	0.295	0.023
Current smoking, n (%)	23 (27.7)	24 (27.0)	1.000	0.017	0.009
Diabetes mellitus, n (%)	25 (30.1)	33 (37.1)	0.420	0.147	0.014
Hypertension, n (%)	46 (55.4)	59 (66.3)	0.161	0.223	0.001
Hyperlipidaemia, n (%)	26 (31.3)	30 (33.7)	0.748	0.051	0.010
Propensity score	0.526 (0.441–0.589)	0.429 (0.351–0.544)	<0.001	—	—

(b) Disease spectrum (not included in the propensity model)

	Unweighted, n (%)			Weighted [2]		
**Outcome** [3]	**Available (n = 83)**	**Not available (n = 89)**	***p*** [1]	**Available, %**	**Not available, %**	**Difference (95% CI)** [4]
Obstructive CAD (≥50%)	30 (36.1)	46 (51.7)	0.047	40.3	45.7	−5.5 (−19.3 to 8.4)
Severe CAD (≥70%)	22 (26.5)	37 (41.6)	0.053	28.4	36.1	−7.7 (−21.0 to 5.8)

Values are median (interquartile range) or n (%); weighted values are percentages. [1] *p*-values were derived from the Mann–Whitney U test for continuous variables and Fisher's exact test for categorical variables. [2] Propensity scores for exercise ECG availability were estimated by logistic regression including age, sex, body mass index, current smoking, diabetes mellitus, hypertension, and hyperlipidaemia (selection model AUC, 0.655). Stabilised inverse-probability-of-testing weights ranged from 0.585 to 2.279 (effective sample size, 158.8). Propensity scores ranged from 0.268 to 0.825 in participants with and from 0.168 to 0.773 in participants without exercise ECG results. [3] Obstructive and severe CAD were defined as ≥50% and ≥70% stenosis on ICA, respectively, and were not included in the propensity model. [4] Weighted difference in percentage points (available minus not available); 95% CIs were derived from 2,000 bootstrap resamples with re-estimation of the propensity model. AUC, area under the receiver operating characteristic curve; CAD, coronary artery disease; CI, confidence interval; ECG, electrocardiography; ICA, invasive coronary angiography; SMD, standardised mean difference.
